# Genetic signatures of polymorphic microsatellite loci in the Ambiguous silver pomfret, *Pampusargenteus* (Teleostei, Stromateidae)

**DOI:** 10.3897/zookeys.810.25602

**Published:** 2018-12-20

**Authors:** Yuan Li, Long-Shan Lin, Tian-Xiang Gao

**Affiliations:** 1 Third Institute of Oceanography, State Oceanic Administration, Xiamen, Fujian 361005, China Third Institute of Oceanography, State Oceanic Administration Xiamen China; 2 Zhejiang Provincial Key Laboratory for Technology Research on Sustainable Utilization of Marine Fishery Resources, Zhoushan, Zhejiang 316021, China Zhejiang Provincial Key Laboratory for Technology Research on Sustainable Utilization of Marine Fishery Resources Zhoushan China; 3 National Engineering Research Center for Marine Aquaculture, Zhejiang Ocean University, Zhoushan, Zhejiang 316022, China Zhejiang Ocean University Zhoushan China

**Keywords:** Genetic diversity, genetic structure, microsatellite DNA, population genetics

## Abstract

*Pampusargenteus* is a broadly exploited pelagic fish species, commonly misidentified as *Pampusechinogaster*. Genetic variation and population structure in *Pampusargenteus* was studied based on seven microsatellite loci. The observed high average allele number, heterozygosity values, and polymorphism information content of *P.argenteus* suggested high genetic diversity. No population genetic differentiation was detected based on the results of pairwise *F*_st_, three-dimensional factorial correspondence analysis (3D-FCA) and STRUCTURE analysis, which implied continuous gene flow. Wilcoxon signed rank tests did not indicate significant heterozygosity excess, and recent genetic bottleneck events were not detected. Coupled with previous mitochondrial DNA results, the findings presented here indicate that high gene flow characterizes the current phylogeographic pattern of the species.

## Introduction

Species of the genus *Pampus* Bonaparte, 1834, are mainly distributed in the Indo-West Pacific Ocean and have a rich landing yield in Kuwait, Iran, India, Malaysia, Thailand, China, Korea and Japan (Jia et al. 2004; Divya et al. 2017). Among these species, *P.argenteus* (Euphrasén, 1788) is a broadly exploited pelagic species that has a high economic value because of its highly appreciated taste. Although all species of *Pampus* are important economical species, the morphological similarity among species of *Pampus* has resulted in considerable confusion in species-level identification. *Pampusargenteus* is the most widely distributed species of the genus, and it is usually identified as *P.echinogaster* (Basilewsky, 1855) because of the morphological similarities (Li et al. 2013, 2017a). This is mainly a consequence of the absence of critical diagnostic morphological characteristics in the description by Euphrasén (1788), based on only one specimen. Li et al. (2013) collected samples of *P.argenteus* from Kuwait, Pakistan, and China and provided updated and improved morphological diagnosis and DNA barcode data. Li et al. (2017a) proposed diagnostic characteristics of *P.echinogaster*, which is significantly different from *P.argenteus*. Therefore, we speculate that *P.argenteus* is a warm-water species that is widely distributed south of the Taiwan Strait and across Indonesia to the Persian Gulf (Yamada et al. 2009; Li et al. 2013). *Pampuspunctatissimus* (Temminck & Schlegel, 1845) was regarded as a synonym of *P.argenteus* by some ichthyologists (Bleeker 1852; Haedrich 1984), while a few researchers recognized differences between these species and provided a redescription of *P.punctatissimus* with a detailed morphological comparison with *P.argenteus* (Liu and Li 1998; Yamada et al. 2009; Nakabo 2013).

*Pampusargenteus* is a multiple batch spawner with indeterminate fecundity, and spawning starts in mid-May and continues until early October. Transformation from the larval to juvenile stage occurs at 40 days after hatching (Almatar et al. 2000). The eggs, larvae, and adults of this species are all pelagic. Although numerous investigations have been performed on *P.argenteus* (Meng et al. 2012; Peng et al. 2010a, 2010b; Zhao et al. 2010, 2011; Wu et al. 2012), many reports could actually be for *P.echinogaster*. Studies on *P.argenteus* mainly focus on its biology (Kuronuma and Abe 1972), reproductive development (Almatar et al. 2004), and resource investigations (Morgan 1985; Pillai and Menon 2000; Narges et al. 2011; Hashemi et al. 2012; Siyal et al. 2013). To date, few population genetic analyses have been conducted with reliable species identification for this species. Although some reports have described *P.argenteus* from the Atlantic-eastern Pacific (Fowler 1938; Davis and Wheeler 1985; Dulčić et al. 2004; Piper 2010; Sami et al. 2014), far from its center of distribution (the western Pacific and Indian Oceans), such identifications should be analyzed further.

Microsatellites (simple sequence repeats, SSRs) are tandemly repeated motifs of 1–6 bases characterized by a high degree of length polymorphism (Zane et al. 2002), and they are sensitive indicators of population genetic structure (Cheng et al. 2015; Song et al. 2016; Stepien et al. 2017). In previous studies, we evaluated the phylogeographical structure of *P.argenteus* by mitochondrial DNA markers, and two lineages were obtained (Li et al. 2017b). To further examine the genetic variation and population structure of *P.argenteus*, seven microsatellite loci were employed in this study, and we aim to infer the relative role of biological characteristics and environmental factors involved in shaping the contemporary population genetic structure of this species by combining the results of mitochondrial DNA.

## Materials and methods

### Sample collection

**Figure 1.**
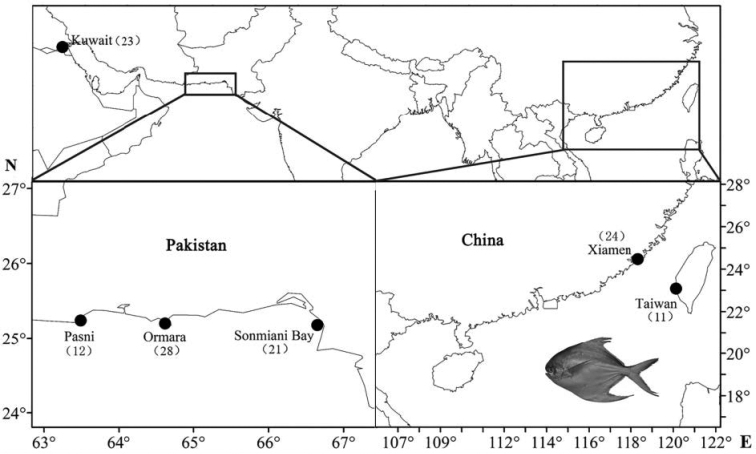
Locations (black circle) for sample collection of *P.argenteus*.

A total of 119 specimens of *P.argenteus* was collected from the coastal waters of Kuwait, Pakistan, and China from 2010 until 2014 (Figure 1, Table 1). All individuals were identified based on morphological characteristics according to Yamada et al. (2009) and Li et al. (2013), and dorsal muscle tissue was excised and preserved in 95% alcohol.

**Table 1.** Summary statistics for the variability seven polymorphic microsatellite loci in six *P.argenteus* populations.

Location	Number of individuals	Date	Locus								Average
			Parameters	Par 03	Par 08	Par 20	Par 05	Par 12	Par 18	Par 17	
Sonmiani Bay (SO)	21	2010.12	*A*	19	12	10	10	15	10	13	12.71
			*R_S_*	13.360	10.000	6.041	5.478	9.412	5.634	10.618	8.649
			*H_O_*	0.429	0.750	0.810	0.476	0.600	0.600	0.789	0.636
			*H_E_*	0.948	0.923	0.855	0.837	0.917	0.844	0.930	0.893
			PIC	0.920	0.892	0.815	0.798	0.885	0.800	0.898	0.858
Ormara (OR)	28	2010.12	*A*	18	15	12	15	16	15	16	15.29
			*R_S_*	11.130	7.396	8.522	10.453	10.721	7.649	10.962	9.548
			*H_O_*	0.333	0.679	0.786	0.857	0.889	0.464	0.778	0.684
			*H_E_*	0.927	0.881	0.899	0.921	0.924	0.885	0.926	0.909
			PIC	0.904	0.852	0.872	0.897	0.899	0.856	0.902	0.883
Pasni (PS)	12	2010.12	*A*	11	9	8	10	14	9	13	10.57
			*R_S_*	9.000	5.647	5.647	6.400	12.522	6.698	8.471	7.769
			*H_O_*	0.250	0.667	1.000	0.750	0.833	0.333	0.667	0.643
			*H_E_*	0.928	0.859	0.859	0.880	0.960	0.888	0.920	0.899
			PIC	0.879	0.805	0.800	0.828	0.914	0.833	0.871	0.847
Kuwait (KW)	23	2011.09	*A*	20	10	11	13	14	15	13	13.71
			*R_S_*	13.444	3.421	6.782	6.541	9.584	8.015	8.015	7.972
			*H_O_*	0.727	0.455	0.957	0.636	0.773	0.478	0.565	0.656
			*H_E_*	0.947	0.724	0.871	0.867	0.916	0.895	0.895	0.874
			PIC	0.921	0.685	0.838	0.834	0.887	0.864	0.864	0.842
Taiwan (TW)	11	2012.09	*A*	14	10	7	11	8	9	11	10.00
			*R_S_*	12.500	5.500	4.172	9.680	7.118	7.333	7.118	7.632
			*H_O_*	0.500	0.727	0.636	0.727	0.455	0.545	0.545	0.591
			*H_E_*	0.968	0.857	0.797	0.939	0.900	0.905	0.900	0.895
			PIC	0.914	0.798	0.732	0.887	0.843	0.848	0.845	0.838
Xiamen (XM)	24	2014.04	*A*	20	16	13	11	16	11	15	14.57
			*R_S_*	15.781	11.755	8.229	6.227	8.417	5.409	9.600	9.345
			*H_O_*	0.417	0.875	0.750	0.708	0.682	0.667	0.708	0.687
			*H_E_*	0.957	0.934	0.897	0.857	0.902	0.832	0.915	0.899
			PIC	0.933	0.909	0.868	0.820	0.872	0.793	0.887	0.869

Abbreviations: *A*: allelic number, *R_S_*: allelic richness, *H_O_*: observed heterozygosity, *H_E_*: expected heterozygosity, PIC: polymorphism information content.

### DNA extraction, amplification and sequencing

Genomic DNA was isolated from muscle tissue by proteinase *K* digestion and extracted using the DNeasy Blood and Tissue Kit (Qiagen, Valencia, CA, USA). Seven microsatellite loci developed by Yang et al. (2006) were used in this study (Table 1). Tailed PCR was used to produce fluorescently labeled DNA fragments (Boutin-Ganache et al. 2001). M13R was added to the 5' end of one primer in each pair. An M13 reverse primer that is fluorescently labeled (FAM, HEX, and TAMRA) was included in the PCR, resulting in a labeled product for detection. All loci were conducted separately in a 25 μL reaction mixture containing 17.25 μL of ultrapure water, 2.5 μL of 10×PCR buffer (including MgCl_2_), 2 μL of dNTPs, 1 μL of fluorescently labeled M13R primer and locus specific primer without tail, 1 μL of locus specific primer with M13 reverse tail, 0.25 μL of Taq polymerase, and 1 μL of genomic DNA (10 ng). All loci were initially screened using the following PCR protocol: 5 min at 94 °C; 35 cycles of 45 s at 94 °C, 45 s at 50~58 °C, and 45 s at 72 °C; and a final step of 15 min at 72 °C. The reactions were then exposed to 72 °C for 45 min and held at 4 °C until further analysis. PCR products were diluted 20 fold with ultrapure Milli-Q water before being further diluted (1 in 5) in formamide containing the LIZ-500 size standard. The samples were separated by capillary gel-electrophoresis on an ABI 3730xl automated sequencer (Applied Biosystems). To score the consistency of microsatellite fragments, nearly 20% of PCR products were restored for replication (Williams et al. 2015). Microsatellite loci genotyping from six populations were determined in GENEMARKER version 2.2.0 software (SoftGenetics, State College, PA, USA).

### Data analysis

The number of alleles (*N_A_*), observed heterozygosity (*H_O_*) and expected heterozygosity (*H_E_*) were estimated using POPGENE 1.32 (Yeh et al. 1999). The polymorphism information content (PIC) was calculated using the Microsoft Excel Microsatellite Toolkit (Botstein et al. 1980; Raymond and Rousset 1995). GENEPOP 3.4 was used to test deviations from the Hardy–Weinberg equilibrium (HWE) and the linkage disequilibrium of each locus (Raymond and Rousset 1995). The presence of null alleles and potential scoring errors were addressed using MICRO-CHECKER 2.2.3 (Van Oosterhout et al. 2004).

FSTAT 2.9.3 (Goudent 2001) was used to calculate the allelic richness (*R*_S_) value and assess the *F*_st_ values. The 3D-FCA (three-dimensional factorial correspondence analysis) was performed in Genetix version 4.05 (Belkir et al. 2004) by making no a priori assumptions about the population groupings. The (δμ)^2^ genetic distance was obtained by POPULATIONS 1.2 (Lespinet et al. 2002), and the UPGMA tree was drawn by Treeview (Page 1996).

The possibility of a cryptic population structure of *P.argenteus* was checked using STRUCTURE (Pritchard et al. 2000). Population groups were simulated from *K*=1 to 6, with each *K* run 10 independent times. Possible mixed ancestry and correlated allele frequencies were assumed, and 1,000,000 Markov chain Monte Carlo (MCMC) steps were used, with the first 100,000 steps discarded as burn-in. To estimate the most likely number of clusters (*K*), an *ad hoc* approach (Pritchard et al. 2000) was performed by obtaining the mean posterior probability of the data *ΔK* and analyzing the dataset for *K*=2, where the value did not increase, peak or plateau, as expected (Li and Liu 2018).

The Bottleneck 1.2.02 program (Piry et al. 1999) was implemented to detect evidence of recent bottleneck events under three mutation models, the infinite allele model (IAM), stepwise mutation model (SMM) and two-phase mutation model (TPM), where 95% single-step mutations and 5% multiple steps mutations with 1000 simulation iterations were set as recommended (Zeng et al. 2012). We also provide a graphical descriptor of the shape about the allele frequency distribution (mode-shift indicator) that differentiates bottlenecked and stable populations (Luikart et al. 1998).

## Results

A total of 150 alleles were detected by seven microsatellite loci for six populations, with a range of 14 (Par 20) to 31 (Par 03) (Table 1). The *N_A_*, *Ho*, *H_E_*, and PIC of *P.argenteus* are shown in Table 1. All the PIC values were greater than 0.5, which suggested the high genetic diversity of this species (PIC>0.5) (Table 1). Two microsatellite loci (Par 03 and Par 05) showed deviations from the Hardy-Weinberg equilibrium in all six populations, and null alleles for these loci were also detected for these two loci. Linkage disequilibrium was not detected between pairs of loci for all populations.

**Figure 2.**
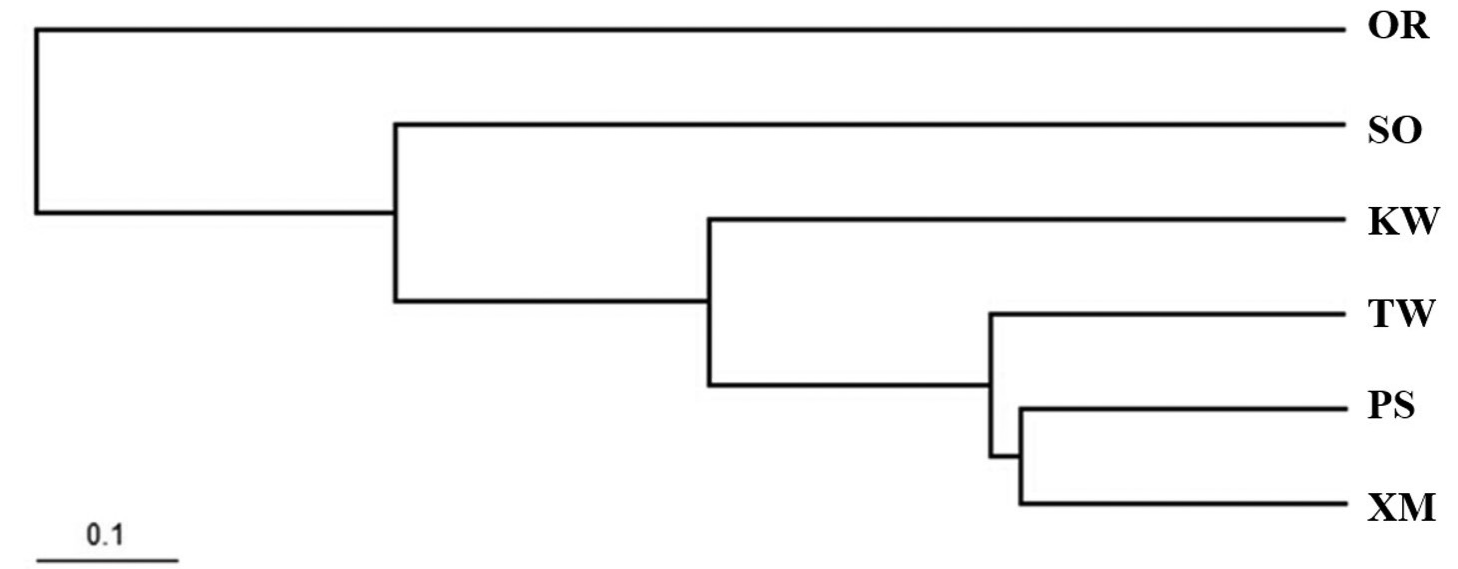
The UPGMA tree based on (δμ)^2^ genetic distance of six *P.argenteus* populations. Abbreviations: SO: Sonmiani Bay, OR: Ormara, PS: Pasni, KW: Kuwait, TW: Taiwan, XM: Xiamen.

The values of pairwise *F*_st_ showed low genetic differentiation among *P.argenteus* populations ranging from 0.001 to 0.026. Most *P*-values were not significant after sequential Bonferroni procedures except those between Kuwait (KW) and the other populations (Xiamen and Sonmiani Bay) (Table 2). The (δμ)^2^ genetic distance was obtained according to the allele frequency by POPULATION software, and the UPGMA tree was constructed by this method (Table 2). The topology of the UPGMA tree showed that *P.argenteus* populations from China, Pakistan and Kuwait coastal waters clustered together and did not relate to their geographical distributions (Figure 2).

**Table 2.** Pairwise *F*_st_ (below diagonal) and (δμ)^2^ genetic distance (above diagonal) among *P.argenteus* populations.

	SO	OR	PS	KW	TW	XM
SO		1.873	1.026	1.267	1.974	0.815
OR	0.005		1.617	1.064	1.530	2.487
PS	0.002	-0.002		0.909	0.505	0.463
KW	0.026 *	0.019	0.018		1.301	1.980
TW	-0.003	0.003	0.001	0.029		1.029
XM	0.004	0.006	0.001	0.022 *	0.010	

*indicate *P*<0.05. Abbreviations: SO: Sonmiani Bay, OR: Ormara, PS: Pasni, KW: Kuwait, TW: Taiwan, XM: Xiamen.

**Figure 3.**
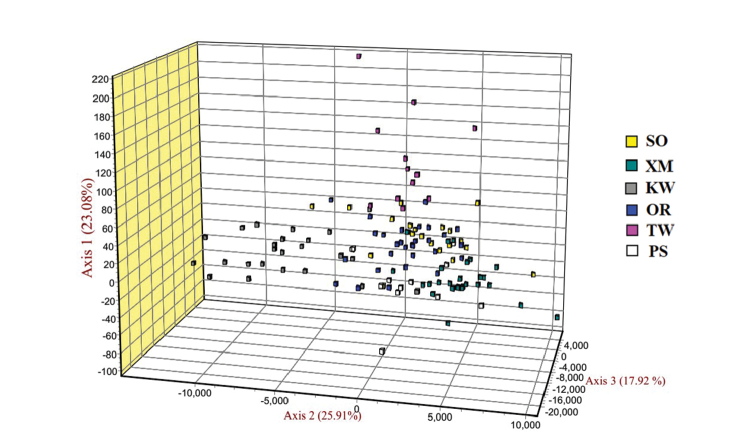
3D-FCA showing relationships among six populations of *P.argenteus* based on seven microsatellite loci. Abbreviations: SO: Sonmiani Bay, OR: Ormara, PS: Pasni, KW: Kuwait, TW: Taiwan, XM: Xiamen.

According to the results of the 3D-FCA, the first, second and third principal components can explain 25.91%, 23.08%, and 17.92% of the overall variation, respectively (Figure 3). Individuals from population Kuwait (KW) and Taiwan (TW) showed a rather distant genetic relationship with the other four populations.

**Figure 4.**
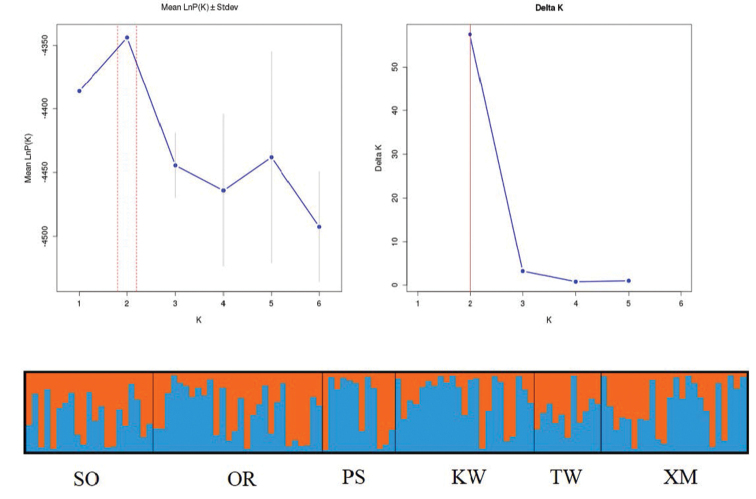
Results of the STRUCTURE analysis from seven microsatellite loci in *P.argenteus* (*K* = 2). Vertical lines are proportional to the probability of individual membership in the simulated cluster. Abbreviations: SO: Sonmiani Bay, OR: Ormara, PS: Pasni, KW: Kuwait, TW: Taiwan, XM: Xiamen.

The Bayesian cluster analysis showed that the model with *K*=2 resulted in the highest *ΔK* value (Figure 4). A total of 70.8% of the sampled individuals from KW were assigned to the second cluster, while five others exhibited lower assignment probabilities to the second cluster (43.2–58.1%). Obvious differences of proportion in the two inferred clusters were not detected in the five other populations (Table 3).

**Table 3.** Proportion of six *P.argenteus* populations in each of the two inferred clusters.

Populations	Inferred clusters		Number of individuals
	1	2	
SO	0.568	0.432	21
OR	0.474	0.526	28
PS	0.419	0.581	12
KW	0.292	0.708	23
TW	0.494	0.506	11
XM	0.420	0.580	24

Abbreviatiosn: SO: Sonmiani Bay, OR: Ormara, PS: Pasni, KW: Kuwait, TW: Taiwan, XM: Xiamen.

The population demography analysis showed no significant heterozygosity excess observed under all three mutation models by the Wilcoxon sign-rank test (*P*>0.05), which suggested that *P.argenteus* should be in mutation-drift equilibrium (Table 4). Additionally, a normal L-shaped allele frequency distribution (‘mode-shift’ indicator) was detected for all six populations, suggesting population stability.

**Table 4.** Results of Wilcoxon’s heterozygosity excess test, Mode shift indicator for a genetic bottleneck in six *P.argenteus* populations.

Populations	Wilcoxon sign-rank test			Mode shift
	IAM	TPM	SMM	
SO	0.004	0.469	0.531	L
OR	0.004	0.531	0.711	L
PS	0.008	0.234	0.469	L
KW	0.148	0.961	0.996	L
TW	0.020	0.289	0.289	L
XM	0.004	0.004	0.945	L

Abbreviations: SO: Sonmiani Bay, OR: Ormara, PS: Pasni, KW: Kuwait, TW: Taiwan, XM: Xiamen.

## Discussion

The degree of genetic variation is particularly important for the sustainability and evolution of species, and the strong correlation between genetic diversity and overall fitness has been reported (Reed and Frankham 2003; Vandewoestijne et al. 2008). Population genetic analyses could provide important insights on the genetic diversity of species and have directly informed fishery managers about the appropriate units of management (Ovenden et al. 2010; Dudgeon et al. 2012). Microsatellites are characterized by a high degree of length polymorphism (Zane et al. 2002), and they represent one of the most popular molecular markers in population genetic studies (Carlsson et al. 2004; Cheng et al. 2015). In this study, high average *N_A_*, heterozygosity values and PIC of *P.argenteus* were detected by seven microsatellite loci, which is consistent with the mitochondrial DNA results of previous studies (Li et al. 2017b). High genetic diversity by mitochondrial DNA and microsatellite DNA may be related to a large effective population size, the immigration of new genes by the intermixing of different populations and/or low selection pressure. Although many marine organisms have been subjected to overfishing, *Pampusargenteus* presents a considerable yield, indicating a large population size. The wide distribution range of habitats indicates that *P.argenteus* faces limited natural selection pressure and can accumulate greater genetic variation. Significant excess *H_O_* was not observed, which showed that *P.argenteus* has not experienced bottleneck effect events. Moreover, the selection of loci with high PIC for the analysis can also lead to high genetic diversity.

Microsatellite markers have demonstrated to be highly sensitive for detecting the population structure of fish (Cheng et al. 2015; Stepien et al. 2017; Li et al. 2018). In this study, analyses based on seven microsatellite loci revealed low levels of genetic differentiation for *P.argenteus*. The Bayesian clustering analysis by STRUCTURE also suggested that the distribution proportions of two inferred clusters were not very different from each other. Similar level of genetic differentiation was detected in mitochondrial DNA (Li et al. 2017b). Marine fish populations usually show fluent gene flow and low levels of genetic differentiation because of their high dispersal potential of different life-history stages coupled with an absence of physical barriers to movement (Beheregaray and Sunnucks 2001). Physical distance has frequently been considered the main factor for isolation (Palumbi 1994). However, although long geographic distances occurred among the three countries, the expected genetic differentiation was not detected. Marine currents may play an important role in shaping the contemporary phylogeographic pattern of marine fishes (Xie and Watanabe 2007). For example, the eggs, larvae, or active adults of *Trachurusjaponicus* can be transported over a long distance by the Kuroshio Current along the shelf slope of the East China Sea from areas northeast of Taiwan to the coastal waters of Japan (Cheng et al. 2015). The migratory behavior of *P.argenteus* during its entire life stage could increase the gene flow and weaken the genetic differentiation among geographic populations (Beheregaray and Sunnucks 2001).

In conclusion, high genetic homogeneity among six *P.argenteus* populations was detected, and the contemporary genetic structure of the species revealed in this study can preliminarily improve the genetic knowledge and provide a firm basis to guide fishery stock management in the Indo-Pacific Ocean. Unfortunately, only six geographical populations of *P.argenteus* were collected, which is not sufficient for an even sampling throughout its entire distribution in the Indo-Pacific Ocean. To describe the phylogeographic pattern of *P.argenteus*, additional representative populations should be collected for further analysis.
